# Successful treatment of trichothiodystrophy with dupilumab

**DOI:** 10.1111/ced.14642

**Published:** 2021-05-06

**Authors:** R. Gruber, A. Zschocke, H. Zellner, M. Schmuth

**Affiliations:** ^1^ Department of Dermatology, Venereology and Allergy Medical University of Innsbruck Innsbruck Austria; ^2^ Department of Pediatrics Pediatrics III Medical University of Innsbruck Innsbruck Austria; ^3^ Department of Pediatrics Pediatrics I Medical University of Innsbruck Innsbruck Austria

## Abstract

Click here for the corresponding questions to this CME article.

Trichothiodystrophy (TTD) is a rare autosomal recessive congenital disorder characterized by neuroectodermal symptoms including sulphur‐deficient brittle hair, dystrophic nails, ichthyosiform erythroderma, growth retardation, intellectual impairment and recurrent infections.^1^, ^2^ In addition, photosensitivity is present in individuals with TTD subtypes 1–3, which are caused by mutations in DNA repair genes encoding subunits of the transcription/repair factor, Transcription factor II human.^2^, ^3^ Treatment is symptomatic and often unsatisfying. We report a patient with TTD successfully treated with dupilumab, a monoclonal antibody that blocks the biologic effects of interleukin (IL)‐4 and IL‐13, which is the first such report to our knowledge.

An 8‐year‐old boy presented for evaluation of his worsening skin condition with severe itching. He had been born at 36 + 3 weeks with a low birth weight and a nonbullous ichthyosiform erythroderma. From toddler age, he had a history of failure to thrive and recurrent respiratory infections, along with brittle hair, dystrophic nails, photosensitivity of eyes and skin, and delayed psychomotor development. In addition, he had allergic rhinoconjunctivitis and asthma requiring long‐term asthma therapy.

Hair analysis revealed trichorrhexis nodosa and a tiger‐tail pattern under polarizing microscopy. Based on the above findings, a clinical diagnosis of TTD, subtype PIBIDS syndrome, was made.

Following informed consent from the parents, genetic analysis was performed, revealed compound heterozygous mutations c.1972C>T (p.Arg658Cys) and c.494_495insT (p.Arg166Alafs*2) in the *ERCC2* gene, confirming the diagnosis.

Despite several topical treatments, including *Vitis vinifera* seed oil, ceramides, glucocorticoids and 0.03% tacrolimus ointment, the patient’s skin condition deteriorated and the increasing pruritus led to insomnia. In addition to dimetindene maleate 1.5 mg (30 drops) three times daily, hydroxysine dihydrochloride 25 mg was given at night to relieve the urge to scratch.

Physical examination showed generalized fine scaling with more prominent coarse scaling on the lower abdomen and the temple areas. Strikingly, in addition to eczema on the eyelids, hands, feet and large flexures, there were prominent scratch marks on the patient’s face and upper back, as well as erosive papules on his arms (Fig. 1a,b).

**Figure 1:**
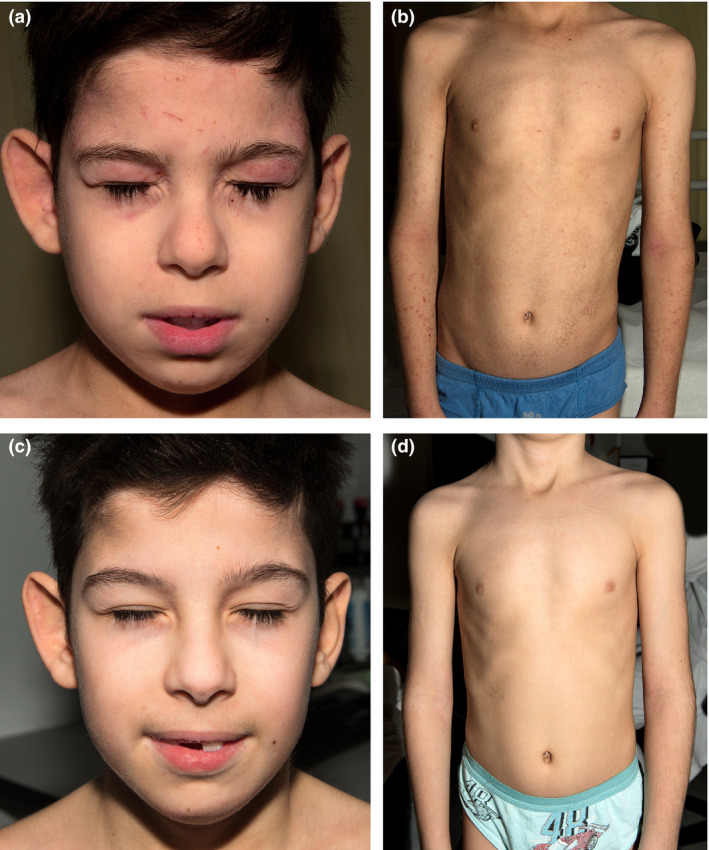
(a,b) Clinical features before treatment: (a) face showing eyelid eczema and scratch marks; (b) trunk and arms with ichthyosis and eczema. (c,d) After 1 year of treatment with dupilumab, (c) the facial skin was no longer inflamed and (d) the skin on the trunk and arms was also healthy.

Informed consent was obtained from the patients to initiate off‐label therapy with dupilumab 200 mg subcutaneously, continued every 2 weeks. Within 3 months, the ichthyosis, eczema and pruritus all significantly subsided. Quality of life improved, with the patient a score of 11 of 30 in the Children's Dermatology Life Quality Index, compared with 28 of 30 prior to treatment with dupilumab. After 1 year of dupilumab therapy, the patient’s skin appeared almost normal (Fig. 1c,d) and his hair and nails were thicker. There was significantly less pruritus and his asthma had gone into remission. No adverse effects were noted. Topical glucocorticoids were needed less frequently and hydroxysine dihydrochloride was reduced to 12.5 mg at night. The boy continues treatment with dupilumab biweekly with sustained efficacy.

Dupilumab is a human monoclonal antibody that binds the shared alpha subunit of the IL‐4 receptor and thus inhibits the signalling of the cytokines IL‐4 and IL‐13. Dupilumab is approved for atopic dermatitis (AD) and asthma, two diseases in which T helper (Th)2 inflammation plays a major role. Skin features of TTD frequently include ichthyosiform erythroderma; however, in approximately 10% of cases there is concurrent eczema, resembling atopic dermatitis.^4^ Although the AD in TTD has not been characterized in detail, it is most likely a Th2‐driven inflammation. In our patient, we initiated off‐label use of dupilumab, which demonstrated rapid onset of action with improvement in scaling and erythema and reduction in itching, allowing us to decrease the use of topical glucocorticoids and antihistamines, which had exacerbated the boy’s learning difficulties. Given the patient’s recurrent respiratory infections and the known beneficial effects of dupilumab on asthma, dupilumab is preferred over immunosuppressants such as ciclosporin or mycophenolate mofetil.

In summary, dupilumab could be a promising novel therapy for TTD; however, further studies are warranted to verify both effectiveness and safety of dupilumab in these patients.
